# Heat shock proteins in hypothermia: a review

**DOI:** 10.3389/fmolb.2025.1564364

**Published:** 2025-05-09

**Authors:** Shang-Jin Song, Guo-Cheng Wu, Li Yi, Xin Liu, Ming-Min Jiang, Xiao-Chen Zhang, Zi-Fei Yin, Wei Gu, Yi Ruan

**Affiliations:** ^1^ School of Traditional Chinese Medicine, Naval Medical University, Shanghai, China; ^2^ Xingcheng Special Duty Sanatorium of Joint Logistics Support Force, Xingcheng, Liaoning, China; ^3^ PLA Naval Medical Center, Shanghai, China

**Keywords:** heat shock proteins, hypothermia, cellular stress, energy metabolism, apoptosis

## Abstract

Hypothermia is a serious condition marked by a significant decrease in core body temperature, posing considerable risks to biological systems. In response to thermal stress, cells activate protective mechanisms, often synthesizing heat shock proteins (HSPs). These highly conserved proteins are crucial in cellular stress responses, primarily functioning as chaperones. HSPs facilitate correct protein folding and prevent misfolding and aggregation, thereby protecting cellular integrity during adverse conditions. This paper explains how HSPs alleviate stress responses related to low body temperature, focusing on energy metabolism, apoptosis, cellular membrane fluidity and stability, and stress signaling pathways. By enhancing cellular repair mechanisms, HSPs help maintain cellular balance and prevent further harm to the organism. Ultimately, the review emphasizes the complex relationship between cellular stress responses and HSPs in hypothermia, highlighting their potential as therapeutic targets for enhancing resistance to the harmful effects of extreme cold exposure. A deeper understanding of these mechanisms could lead to strategies that improve survival rates in hypothermic patients. It may also reveal ways to modulate HSPs’ activity for enhanced cellular protection.

## 1 Introduction

Hypothermia is defined as an abnormally low body temperature, usually below 35°C (95°F) (Okumura et al., 2025). Hypothermia is relatively uncommon in the general population. However, its occurrence has been increasing, especially among individuals involved in cold-exposure activities such as diving, maritime operations, and mountaineering (Paal et al., 2022). Clinically, hypothermia shows various symptoms, including shivering, confusion, and lethargy. In severe cases, hypothermia can cause loss of consciousness, irregular heartbeats, and even death, complicating the care of affected individuals (Bjertnaes et al., 2022).

Heat shock proteins (HSPs), also called molecular chaperones or stress proteins, are highly conserved proteins produced by cells in response to increased temperatures and various biological and environmental stressors, such as rapid temperature shift, oxidative stress, and infections (Hagymasi et al., 2022; Singh and Prasad, 2022). HSPs are essential for maintaining cellular balance by ensuring proper protein folding, transport, and synthesis; furthermore, they prevent the misfolding and clumping of newly formed and stress-damaged proteins, stabilize protein structures, assist in directing proteins to their correct locations, and maintain overall protein quality control (Chen et al., 2018).

HSPs are essential for maintaining proteins and are also crucial in several key cellular processes, including regulating the energy metabolism, aiding intracellular signaling, and controlling apoptosis (Bankapalli et al., 2015; Kassis et al., 2021; Yang et al., 2022; Kuppuswami and Senthilkumar 2023; Johnson and Sarosiek, 2024). The various roles of HSPs underscore their significance in both physiological and pathological conditions. Recent studies have revealed the crucial roles of HSPs during hypothermia, demonstrating that they offer vital protective and recovery mechanisms when the body experiences cold stress (Lee et al., 2017; Singh and Prasad, 2022). As temperatures decrease, the likelihood of protein misfolding rises, highlighting the critical importance of HSPs’ protective functions. This review will examine the roles and regulatory mechanisms of HSPs in hypothermia. It will emphasize their importance in understanding and possibly alleviating the impacts of this condition.

## 2 Hypothermia

The epidemiological profile of hypothermia is intriguing; although it is rare in the general population, awareness of the condition has been increasing across different regions. In European countries and New Zealand, the annual incidence rates of hypothermia vary from 0.13 to 6.9 cases per 100,000 people (Herity et al., 1991; Taylor et al., 1994; Vassal et al., 2001; Brandstrom et al., 2014; Paal et al., 2018). In contrast, many developing nations lack comprehensive data on the incidence of hypothermia, highlighting a significant gap in both epidemiological research and public awareness in these regions. Hypothermia is clinically classified into three levels: mild (32°C–35°C), moderate (28°C–32°C), and severe (<28°C), with each level linked to progressively serious physiological disturbances (Paal et al., 2018).

The pathophysiological mechanisms of hypothermia involve complex bodily responses to lower temperatures, which can result from environmental factors like getting lost, avalanches, heavy snowfall, substance abuse, or falling from heights, as well as from immersion in cold water due to drowning or maritime accidents (Bjertnaes et al., 2022; Paal et al., 2022). These situations result in significant heat loss due to radiation, conduction, and convection. Furthermore, impaired thermogenesis or thermoregulation can exacerbate hypothermia (Kyokan et al., 2023; Savioli et al., 2023). This may lead to metabolic disruptions that can cause rapid death. Clinically, individuals with hypothermia may exhibit symptoms that range from mild, such as shivering and confusion, to severe, including lethargy, loss of consciousness, and cardiovascular failure (Bjertnaes et al., 2022; Paal et al., 2022).

Contemporary researches on hypothermia primarily focuses on its effects at both systemic and organ-specific levels (Bjertnaes et al., 2022). When a person experiences hypothermia, the body activates several mechanisms to conserve heat, such as narrowing peripheral blood vessels (vasoconstriction), generating heat through muscle contractions (shivering), and utilizing brown adipose tissue along with metabolic processes for thermogenesis. If these physiological responses are insufficient to maintain a normal body temperature, the patient’s functional abilities begin to decline as the core temperature drops, leading to a state of functional suppression that impacts various organs in different ways. For instance, in individuals with hypothermia, the metabolic rate decreases by about 6%–7% for each degree Celsius drop in body temperature (Savioli et al., 2023). Additionally, as the core temperature falls, heart rate and vascular resistance increase, which in turn raises blood pressure. However, if the body temperature continues to drop, especially below 32°C, cardiovascular function progressively deteriorates, resulting in physiological bradycardia, a sign of cardiac depression (Bjertnaes et al., 2022; Kyokan et al., 2023).

This article primarily discusses cellular level changes. When cells encounter cold stimuli, they undergo cellular stress, initiating a series of defensive responses. These responses encompass energy metabolism, programmed cell death, and cell membrane stability, aimed at maintaining cell survival, repairing damage, or eliminating damaged cells through programmed death.

### 2.1 Energy metabolism

To adapt to the effects of hypothermia, the body’s initial response is to boost heat production, which consequently disrupts cellular energy metabolism. When exposed to acute cold, energy consumption rises, and the rate of glycolysis increases to satisfy the energy needs of cells (Liu et al., 2021). This cold stimulation also enhances the oxidation of fatty acids in brown adipose tissue, influencing lipid metabolism, increasing heat production, and aiding in the maintenance of body temperature (Nishiyama et al., 2022; Straat et al., 2022).

Mitochondria, often referred to as the “power plants” of cells, are essential for energy metabolism as they produce ATP through oxidative phosphorylation, the main source of cellular energy. The impact of cold environments on mitochondria is complex. Studies indicate that cold exposure leads to notable alterations in mitochondrial function, stimulating both mitochondrial activity and biogenesis (Sugasawa et al., 2016). However, during acute cold stress, mitochondrial energy metabolism can be suppressed, resulting in insufficient energy supply for cells, which may lead to cell apoptosis and dysfunction (Nakamura et al., 2021; Iuchi et al., 2023). Additionally, cold exposure heightens oxidative stress within mitochondria, causing further damage to their structure and function. Specifically, cold stimulation can harm mitochondrial DNA and increase the permeability of the mitochondrial membrane (Lindgren and Barnard, 1972; Nishimura and Watanuki, 2014), thereby compromising mitochondrial integrity and functionality (Park et al., 2019).

### 2.2 Cellular apoptosis

Hypothermia not only induces cell apoptosis but also significantly impacts cell survival and function through various signaling pathways and biological mechanisms. Cold-induced oxidative stress activates crucial components of the MAPK pathway, such as ERK, JNK, and p38 MAPK, which play vital roles in regulating cell growth, differentiation, and apoptosis. For example, exposure to cold can increase the levels of reactive oxygen species (ROS) within cells, creating a state of oxidative stress that promotes apoptosis by activating the p38 MAPK pathway (King et al., 2024).

Additionally, under cold conditions, the phosphorylation level of Akt decreases, which diminishes the cell’s ability to respond to survival signals, ultimately leading to a higher rate of apoptosis. In cardiac cells specifically, cold exposure inhibits the activity of the PI3K/Akt pathway, contributing to increased apoptosis and dysfunction (Yi et al., 2022). Furthermore, cold stimuli can result in the upregulation of pro-apoptotic protein Bax and the downregulation of anti-apoptotic protein Bcl-2, further enhancing the process of cell apoptosis (Yi et al., 2022).

### 2.3 Cellular membrane

The cell membrane acts as a protective barrier for cells and is mainly made up of phospholipids, cholesterol, and glycolipids. When temperatures drop, the lipid bilayer of the cell membrane can become more compact, potentially transitioning from a liquid to a solid state. This change can significantly affect the fluidity and stability of membrane. A reduction in fluidity may disrupt the function and distribution of membrane proteins, which in turn can influence cell signaling and the transport of materials (Magee et al., 2005; Gusain et al., 2023).

Researches show that when exposed to cold, cells increase the proportion of unsaturated fatty acids in their membranes to help maintain fluidity. For example, some plants boost their levels of unsaturated fatty acids, like linoleic and linolenic acids, in response to low temperatures, which helps enhance both fluidity and stability of their membranes (Sanchez Granel et al., 2019; Ahad et al., 2023). Moreover, cells might also ramp up cholesterol production in cold conditions to further support membrane fluidity, thereby shielding themselves from damage caused by the cold (Cao et al., 2024).

The impact of low temperatures on membrane fluidity can also affect the ion balance and signal transduction of cell, by changing the membrane potential and the functioning of ion channels (Li W. et al., 2023). Cold exposure activates certain signaling molecules that are vital for regulating membrane fluidity. For instance, an increase in calcium ions can lead to modifications in membrane fluidity, which subsequently influences cellular metabolism and various physiological functions (Jahed et al., 2023).

## 3 Heat shock proteins

HSPs are conserved molecular chaperones found in all life forms, essential for protein folding and cellular protein balance. HSPs are classified into families according to their molecular weight, including HSP110, HSP90, HSP70, HSP60, HSP40, and small heat shock proteins (sHSPs). HSPs protect cell integrity by preventing protein aggregation. They also facilitate the repair and degradation of misfolded proteins. Additionally, HSPs play a key role in regulating various biological processes, such as cell signaling, programmed cell death (apoptosis), energy metabolism, and membrane integrity (Sadura et al., 2020; De Maio and Hightower, 2021; Hagymasi et al., 2022; Singh and Prasad, 2022; Xin et al., 2022; Singh et al., 2024).

### 3.1 HSP110

The HSP110 consists of two key members: the heat shock-induced HSP110 and Grp170, which is mainly activated by glucose deprivation in the endoplasmic reticulum (Yakubu and Morano, 2018). HSP110 plays a vital role in protecting proteins by preventing their aggregation during heat stress. It is essential for the major protective and repair mechanisms that deal with denatured proteins and contribute to heat tolerance in mammalian cells. Acting as a nucleotide exchange factor for HSP70 (like HSPA4), HSP110 helps release substrates from HSP70, which boosts the ability of HSP70 to manage large macromolecular substrates (Michel et al., 2015). Additionally, HSP110 stabilizes partially folded intermediates through its C-terminal domain. The specific substrates that HSP110 targets include large molecular complexes, such as microtubules and ribosomal subunits, as well as certain proteins prone to aggregation, like TDP-43 and superoxide dismutase 1 (Xu et al., 2012; Chakafana and Shonhai, 2021).

### 3.2 HSP90

HSP90 is another important heat shock protein involved in folding and stabilizing key signaling proteins and transcription factors. Its effectiveness is determined by a complex structure and dynamic conformational changes, enabling interactions with diverse co-chaperone proteins that influence the function of various target substrates, including kinases such as EGFR, HER2, RAF, AKT, and CDK4; nuclear receptors like glucocorticoid receptor, estrogen receptor, and androgen receptor as well as transcription factors such as p53, HIF-1α, and NF-κB (Varisco et al., 2009; Oranges et al., 2024; Silbermann et al., 2024). The binding and breakdown of ATP are crucial for the function of HSP90, as these processes cause structural changes in substrates, facilitating their activation (Genest et al., 2019; Silbermann et al., 2024). Currently, extensive research focuses on HSP90 in cancer biology, where it has become a key therapeutic target for anti-cancer treatments (Liew et al., 2022).

### 3.3 HSP70

The HSP70 family of heat shock proteins are essential for maintaining cellular stability. HSP70 consists of two primary regions: the N-terminal domain that binds nucleotides and the C-terminal domain that interacts with substrate proteins, which include newly synthesized polypeptide chains released from ribosomes and specific signaling molecules such as Bcl-2 family proteins and apoptosis-related proteins (Park et al., 2017; Wang et al., 2020; Ambrose et al., 2024). HSP70 facilitates proper protein folding by binding to them and releasing them in a manner dependent on ATP (Genest et al., 2019). This action helps prevent protein aggregation. It is also essential for repairing misfolded proteins through refolding mechanisms, which ensures the correct functioning of intracellular proteins. Furthermore, HSP70 collaborates with various co-chaperone proteins to support protein quality control and degradation processes (Kiraly et al., 2020; Zhang et al., 2023).

### 3.4 HSP60

HSP60 is mainly found in the cytosol and mitochondria, but it can also be detected on the cell surface. As the primary ATP-dependent chaperone in mitochondria, HSP60 is one of the most abundant proteins in these organelles and is essential for maintaining the stability of mitochondrial proteins (Comert et al., 2024). Its key substrates consist of mitochondrial matrix proteins, including pyruvate dehydrogenase and ornithine transcarbamylase, which are vital for various metabolic processes (Singh et al., 2024).

### 3.5 HSP40

The HSP40 protein family, commonly referred to as DNAJ proteins, plays a crucial role as co-chaperones that help regulate the activity of HSP70 (Zarouchlioti et al., 2018). They achieve this by recognizing and binding to specific substrates, which in turn stimulates the ATPase activity of HSP70 (Kumar and George, 2013). Together, HSP40 and HSP70 form an essential chaperone system that aids in the proper folding of polypeptides in both prokaryotic and eukaryotic organisms (Zhu et al., 2019). HSP40 specifically targets substrates that are rich in hydrophobic regions, including newly synthesized secretory proteins like proinsulin and improperly folded glycoproteins, such as those that are incompletely glycosylated within the endoplasmic reticulum (Faust et al., 2020).

### 3.6 sHSPs

sHSPs are low molecular weight proteins that prevent substrate protein aggregation, crucial for protecting cells from stress-induced damage. sHSPs can form dynamic oligomers through their α-crystallin domains, allowing them to bind to exposed hydrophobic patches. Their primary substrates are partially folded intermediate proteins (Raju and Abraham, 2011; Clark et al., 2012). sHSPs are vital during cellular responses to thermal stress, oxidative stress, and environmental challenges, underscoring their role in maintaining cellular integrity (Wu J. et al., 2022). Besides their protective functions, sHSPs regulate apoptosis, promote autophagy, and stabilize the cytoskeleton (Mogk and Bukau, 2017; Wu Y. et al., 2022). Their capacity to reduce protein aggregation and enhance cell survival under stress highlights the critical role of sHSPs in boosting cellular resilience. Consequently, the significance of sHSPs in cellular stress responses and related diseases has gained increasing attention from researchers (Gu et al., 2023).

## 4 Heat shock transcription factor (HSF)

HSF family shows significant diversity among eukaryotes, with differences in the number and roles of its members across various species. HSF1 is a key player in the stress response, activating HSPs like HSP70 and HSP90 when faced with stresses such as heat, oxidative stress, and hypoxia; it is widely expressed and significantly upregulated during stress (Takii et al., 2024). HSF2, on the other hand, plays a crucial role in developmental processes, including embryogenesis and brain development, and works alongside HSF1 to co-regulate specific target genes, showing high expression levels in embryonic tissues and germ cells (Santopolo et al., 2021). In avian species, HSF3 acts as the main heat stress factor, but it has lost some of its functions in mammals, where it may still contribute to stress responses in certain tissues, like the intestines, being more prominently expressed in birds than in mammals (Fujimoto et al., 2010). HSF4 is involved in lens differentiation by activating α-crystallin and also plays a part in neurodevelopment; it has two splice variants, HSF4a, which inhibits, and HSF4b, which activates, and is specifically expressed in the lens, brain, and skin (Gao et al., 2017). Lastly, the function of HSF5 is not well understood, but it may be linked to germ cell development, as it is primarily expressed in the testes and ovaries (Liu et al., 2024).

HSFs are crucial for regulating HSP genes. When cells face stress, HSFs activate heat shock genes. They do this by binding to specific sequences called heat shock elements found in the promoters of HSP genes (Znaidi, 2024). In response to proteotoxic stress, HSF1 forms a transcriptional complex that interacts with chromatin changes, enhancing the expression of HSP genes (Fujimoto et al., 2023). Additionally, when environmental temperatures drop, cells trigger their temperature-sensing mechanisms, activating HSFs and increasing their transcriptional activity. These processes allow HSFs to help cells adapt to thermal stress and significantly enhance cellular defenses against proteotoxicity. This underscores their critical role in managing low-temperature exposure and various cellular stress responses.

## 5 HSPs in hypothermia

Initial investigations indicate that cold stress can increase HSP70 expression in myocardial cells by six times compared to standard control conditions (Laios et al., 1997). This increase in HSP70 enhances cellular tolerance to various stressors, improving cell survival rates under adverse conditions (Bukau and Horwich, 1998; Li Y. et al., 2023; Deng et al., 2024). Cullen found a significant increase in nuclear levels of heat shock protein A12A, a newly identified member of the HSP70 family, in white adipose tissue after 5 days of cold exposure in mice (Cheng et al., 2019). Additionally, Farashi showed that stem cells can reduce oxidative stress and improve their survival rates by secreting HSPs in low-temperature conditions (Farashi and Sharifi, 2021).

**FIGURE 1:**
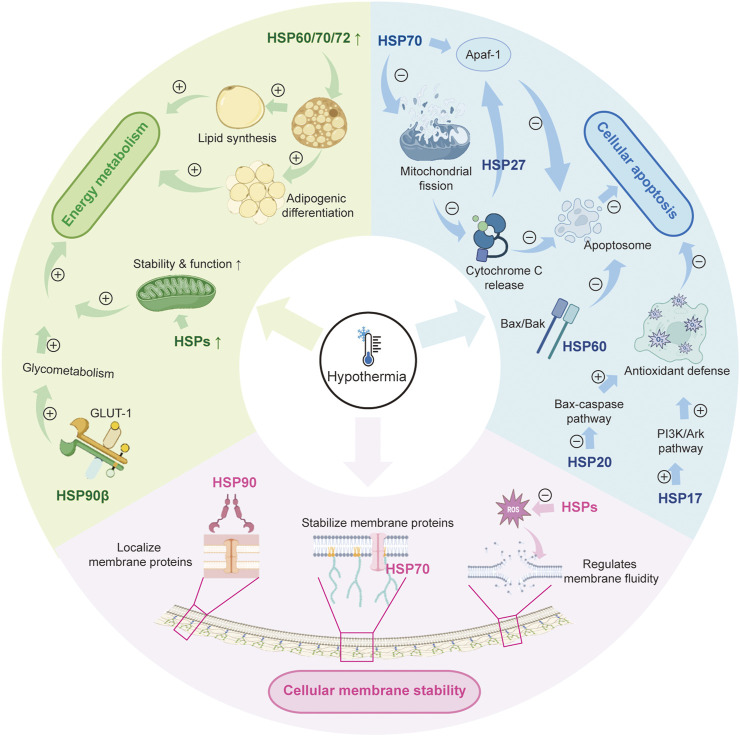
The role of HSPs in hypothermia. HSP: heat shock protein; GLUT-1: glucose transporter 1; PI3K/AKT: phosphatidylinositol 3-kinase/protein kinase B.

More research continues to elucidate the multifaceted regulatory network between hypothermia and HSPs, particularly in cellular energy metabolism, apoptotic regulation, and membrane stabilization (Figure 1). Hypothermia triggers the expression of HSPs and also affects their function by interacting with different signaling pathways (Cuthbert et al., 2019; Gergs et al., 2021; Mishra, 2022). This intricate relationship underscores the crucial role of HSPs in the cellular response to low-temperature stress and highlights their potential as therapeutic targets for hypothermia-related conditions.

The AKT signaling pathway is crucial for regulating HSPs, especially HSP70, in astrocytic cells. AKT directly enhances the expression of HSP70 and also activates HSF1, which increases HSP70 levels necessary for cellular protection (Ren et al., 2020). When cells encounter stressful conditions, HSP90 plays a role in activating the PKM2-AKT signaling pathway, which helps prevent cell death by promoting HSP70 expression, a process that HSF1 regulates. HSP70 is vital for the molecular chaperone functions of HSP90 (Chen et al., 2020). Additionally, when HSP70 is released into the extracellular space, it helps protect cells from damage caused by stress (Chen et al., 2020; Zhang et al., 2020).

Experiments designed to induce stress in HT29 colorectal cancer cells have demonstrated that the MAPK signaling pathway is essential for enhancing the expression of HSPs (Yahya et al., 2024). Additionally, studies have shown that the overexpression of HSP20 inhibits both the MAPK and AKT signaling pathways. This inhibition leads to decreased phosphorylation levels of critical proteins such as AKT, ERK, JNK, and p38 MAPK. Conversely, the lack of HSP20 produces an opposing effect, underscoring its regulatory role within these pathways (Yang et al., 2022). Furthermore, external stimuli can activate p38 MAPK and AKT signaling through ROS, which in turn activate HSF1. The activation of HSF1 results in the increased production of HSPs, thereby offering protection to the cells (Liu et al., 2022).

### 5.1 Regulation of HSPs in energy metabolism

In cold environments, cells activate metabolic processes to increase energy production and raise their temperature. Mitochondria are the central hub of cellular energy metabolism, responsible for synthesizing ATP and producing metabolic intermediates. HSPs are crucial for supporting mitochondrial function by preventing damage through various mechanisms. For example, HSPs stabilize mitochondrial membranes and reduce oxidative stress. This stabilization helps maintain mitochondrial performance and ensures a steady energy supply for the cell (Roundhill et al., 2016). When cells are stressed, HSP levels increase significantly, which is essential for reducing mitochondrial injury and enhancing cell survival (Chang et al., 2022; Kuppuswami and Senthilkumar 2023). Additionally, HSPs aid in mitochondrial biogenesis and autophagy, further enhancing mitochondrial health and functionality (Zhang and Dong, 2021). HSPs are essential for maintaining calcium balance in mitochondria and for repairing and transcribing mitochondrial DNA, which support cellular energy metabolism (Wen et al., 2021; Ding et al., 2022; Lu et al., 2022).

Through a paracrine mechanism, HSPA12A acts as a novel regulator of WAT browning, as evidenced by HSPA12A^−/−^ mice exhibiting attenuated body temperature drop and enhanced thermogenic gene expression compared to WT mice after cold exposure, highlighting its crucial role in low-temperature energy metabolism (Cheng et al., 2019). Additionally, HSP31 plays a crucial role in maintaining intracellular levels of glutathione and nicotinamide adenine dinucleotide phosphate. These molecules help protect against oxidative stress. During oxidative stress, HSP31 relocates to the mitochondria, offering protective benefits to this organelle (Bankapalli et al., 2015).

Furthermore, HSPs are involved in carbohydrate and lipid metabolism; for instance, HSP90β directly interacts with the key glucose transporter 1 to promote the glycolytic pathway (Yang et al., 2024). In lipid metabolism, HSP60, HSP70, and HSP72 facilitate adipogenic differentiation and lipid synthesis (Mei et al., 2021; Shin and Ajuwon, 2023; Hu et al., 2024).

Overall, HSPs play a crucial role in regulating energy metabolism and preserving mitochondrial integrity, especially during low-temperature stress. They facilitate both carbohydrate and lipid metabolism, which helps meet the energy demands of cells while also improving resistance to oxidative damage.

### 5.2 Regulation of HSPs in cellular apoptosis

Apoptosis, commonly known as programmed cell death, is a highly regulated process that involves various signaling pathways. During hypothermia, cells may initiate apoptosis to eliminate dysfunctional ones (Chen et al., 2022). In this scenario, the permeability of the mitochondrial membrane increases, allowing pro-apoptotic proteins like cytochrome c to move into the cytosol. Once in the cytosol, cytochrome c binds to Apaf-1 and procaspase-9, leading to the formation of the apoptosome, which subsequently triggers a cascade of proteolytic enzymes (Johnson and Sarosiek, 2024). HSP70 plays a protective role by inhibiting mitochondrial fission, thereby reducing mitochondrial dysfunction and suppressing the apoptotic pathway (Hu et al., 2022). Furthermore, HSP70 directly interacts with Apaf-1, providing an additional mechanism to inhibit apoptosis (Garrido et al., 2006). Similarly, HSP27 protects cells from various apoptotic signals by binding to cytochrome c, which prevents its interaction with Apaf-1 and procaspase-9, thereby inhibiting the formation of the apoptosome (Nagaraja et al., 2012; Bruey et al., 2000).

HSP70 also reduces apoptosis of *Marsupenaeus japonicus* under low-temperature stress, whereas HSP70 knockout significantly increased mortality and upregulated apoptosis-related genes (e.g., Caspase3 and Bcl-2) in *M. japonicus* under cold stress (Ren et al., 2025). Similarly, HSP20 provides protection against apoptosis during ischemia-reperfusion events by inhibiting the Bax-caspase pathway (Martin et al., 2014). Additionally, HSP17 activates the PI3K/Akt signaling pathway, which helps reduce oxidative stress-related damage and apoptosis in H9c2 cardiomyocyte cells, enhancing their ability to withstand environmental stressors (Chen et al., 2022). Furthermore, HSP60 interacts with Bax and Bak proteins to form aggregates, effectively preventing apoptosis that these apoptotic factors would otherwise induce (Kirchhoff et al., 2002).

Furthermore, HSPs are crucial in regulating apoptosis by influencing the cleavage and activation of caspase precursors. This regulatory mechanism is observed in various disease states, particularly in cancer and neurodegenerative diseases, where the upregulation of HSP expression is often associated with reduced caspase activity (Ahn et al., 2024). HSPs help boost the cell’s antioxidant defenses while lowering the levels of ROS, which in turn reduces the occurrence of apoptosis (Kassis et al., 2021). Moreover, when cells experience stress, the upregulation of HSPs activates protective mechanisms that enhance cellular resistance to apoptotic signals (Singh et al., 2024).

### 5.3 Regulation of HSPs in cellular membrane

The cell membrane plays a vital role as a protective barrier, allowing for the transport of substances, the transmission of signals, and communication between cells. Maintaining its structural integrity and functionality is essential for cell survival. Research has shown that HSPs have lipid-binding and membrane-interacting properties that are crucial for the transport, proper folding, and localization of membrane proteins. For example, HSP90 is key in localizing membrane proteins by directly binding to them (Rogala-Koziarska et al., 2019). Similarly, HSP70 acts as a chaperone for these proteins, helping to stabilize the cell membrane during stressful situations, such as low temperatures (Balogi et al., 2019). Additionally, in plant cells, HSP70 has been observed to interact with phospholipase Dδ, with this interaction becoming significantly stronger under heat stress (Song et al., 2021). This interaction plays an important role in regulating membrane fluidity and stability. For example, SIHSP17.7, a kind of sHSPs, modulates tolerance to cold via membrane fluidity (Wu Y. et al., 2022).

HSPs play a crucial role in influencing the fluidity of cell membranes by regulating the production of ROS and the related signaling pathways. Research has demonstrated that membrane fluidity is closely linked to ROS generation; an increase in membrane fluidity can reduce ROS formation, thereby alleviating heat stress-induced damage to cells (Mironov et al., 2019). Consequently, HSPs are not only involved in direct interactions with membrane proteins but also participate in a complex signaling network that regulates membrane fluidity. This function of HSPs is vital for maintaining membrane stability and ensuring cellular functionality when faced with environmental stressors.

## 6 Conclusion

The critical role of HSPs in mitigating cellular damage during hypothermia is becoming increasingly clear. This review emphasizes their diverse contributions to stress adaptation, regulation of apoptosis, stabilization of membranes, and energy metabolism. HSPs help maintain mitochondrial function, inhibit apoptotic pathways, and preserve membrane fluidity, making them key players in cellular resilience when exposed to low temperatures. These functions highlight not only the evolutionary importance of HSPs in maintaining protein stability but also their potential therapeutic benefits in improving survival rates for patients experiencing hypothermia.

However, researches on HSPs in the context of hypothermia is still limited, primarily focusing on systemic or organ-level effects rather than the direct cellular and molecular mechanisms at play. Most studies referenced rely on indirect evidence that connects HSP functions to cold-induced cellular disruptions, such as energy deficits, apoptosis, and membrane instability, due to a lack of direct investigations into HSP dynamics in hypothermic conditions. While this approach is useful for generating hypotheses, it carries inherent limitations, including the risk of overlooking specific regulatory networks that vary by context. Additionally, the interactions between HSPs and signaling pathways like AKT, MAPK, and STAT need further exploration in hypothermia-specific contexts to better understand their causal relationships and relevance for clinical applications.

Future studies should focus on developing *in vivo* and *in vitro* models that replicate hypothermic conditions to better understand how HSPs are induced, how they undergo post-translational modifications, and how they interact with pathways sensitive to cold. Additionally, investigating how HSP responses vary in different tissues and how these responses are regulated over time as hypothermia progresses could lead to improved therapeutic strategies. New approaches to modulating HSPs, including pharmacological enhancers or gene-editing techniques, may provide innovative ways to strengthen cellular defenses against damage caused by cold exposure.

In summary, while this review highlights the protective functions of HSPs in hypothermia, it also emphasizes the need for focused research on the underlying mechanisms. Addressing these gaps will enhance our understanding of how cells adapt to cold and could lead to clinical applications that improve outcomes for conditions related to hypothermia.
